# Feasibility and safety of high-dose adenosine perfusion cardiovascular magnetic resonance

**DOI:** 10.1186/1532-429X-12-66

**Published:** 2010-11-16

**Authors:** Theodoros D Karamitsos, Ntobeko AB Ntusi, Jane M Francis, Cameron J Holloway, Saul G Myerson, Stefan Neubauer

**Affiliations:** 1 Department of Cardiovascular Medicine, University of Oxford, John Radcliffe Hospital, UK

## Abstract

**Introduction:**

Adenosine is the most widely used vasodilator stress agent for Cardiovascular Magnetic Resonance (CMR) perfusion studies. With the standard dose of 140 mcg/kg/min some patients fail to demonstrate characteristic haemodynamic changes: a significant increase in heart rate (HR) and mild decrease in systolic blood pressure (SBP). Whether an increase in the rate of adenosine infusion would improve peripheral and, likely, coronary vasodilatation in those patients is unknown. The aim of the present study was to assess the tolerance and safety of a high-dose adenosine protocol in patients with inadequate haemodynamic response to the standard adenosine protocol when undergoing CMR perfusion imaging.

**Methods:**

98 consecutive patients with known or suspected coronary artery disease (CAD) underwent CMR perfusion imaging at 1.5 Tesla. Subjects were screened for contraindications to adenosine, and an electrocardiogram was performed prior to the scan. All patients initially received the standard adenosine protocol (140 mcg/kg/min for at least 3 minutes). If the haemodynamic response was inadequate (HR increase < 10 bpm or SBP decrease < 10 mmHg) then the infusion rate was increased up to a maximum of 210 mcg/kg/min (maximal infusion duration 7 minutes).

**Results:**

All patients successfully completed the CMR scan. Of a total of 98 patients, 18 (18%) did not demonstrate evidence of a significant increase in HR or decrease in SBP under the standard adenosine infusion rate. Following the increase in the rate of infusion, 16 out of those 18 patients showed an adequate haemodynamic response. One patient of the standard infusion group and two patients of the high-dose group developed transient advanced AV block. Significantly more patients complained of chest pain in the high-dose group (61% vs. 29%, p = 0.009). On multivariate analysis, age > 65 years and ejection fraction < 57% were the only independent predictors of blunted haemodynamic responsiveness to adenosine.

**Conclusions:**

A substantial number of patients do not show adequate peripheral haemodynamic response to standard-dose adenosine stress during perfusion CMR imaging. Age and reduced ejection fraction are predictors of inadequate response to standard dose adenosine. A high-dose adenosine protocol (up to 210 mcg/kg/min) is well tolerated and results in adequate haemodynamic response in nearly all patients.

## Introduction

First pass perfusion cardiovascular magnetic resonance (CMR) is routinely performed under vasodilatory pharmacological stress with either adenosine or dipyridamole [1,2]. Adenosine is the most widely used vasodilator agent because it is safe, well tolerated, and easily controlled [3]. It generates systemic vasodilatation and reflex sympatho-excitation with consequent mild decrease in systolic blood pressure, slight increase in heart rate, and modest increase in double product [4]. As a potent coronary vasodilator, adenosine causes up to a 4-fold increase in myocardial blood flow in areas supplied by normal coronary arteries. In contrast, in the presence of epicardial coronary stenoses, flow inhomogeneities give rise to regional perfusion defects during the first pass of a gadolinium based contrast [1].

With the standard adenosine dose of 140 mcg/kg/min most, but not all, patients develop maximal vasodilatation [4-6]. In patients with no or mild signs of peripheral vasodilatation however, the adequacy of coronary vasodilatation is questioned. Whether this inadequate cardiovascular response would respond to an increase in the rate of adenosine infusion is unknown. It is also unclear whether a high-dose adenosine infusion protocol is safe and would be tolerated by patients undergoing stress perfusion CMR. We hypothesized that an increase in the adenosine infusion rate up to 210 mcg/kg/min would result in an improved peripheral haemodynamic response in perfusion CMR subjects who failed to show characteristic changes in blood pressure and heart rate with the standard adenosine dose (140 mcg/kg/min).

## Methods

The study population consisted of patients with known or suspected coronary artery disease (CAD) who underwent adenosine stress perfusion CMR imaging for clinical purposes. All patients gave informed consent before the CMR scan. All subjects were initially screened for the presence of contraindications to adenosine, which include asthma, unstable angina or acute myocardial infarction within two weeks of the study, 2^nd ^or 3^rd ^degree atrioventricular (AV) block and bifascicular block. A 12-lead ECG was performed before the CMR scan. All patients were asked to abstain from caffeine for at least 12 hours prior to the scan. However, patients who reported caffeine intake within the last 12 hours were still included in the study. Prior to the CMR scan, a physician carefully explained the procedure to subjects, with emphasis on potential adenosine-related symptoms. Subjects were continuously monitored with peripheral oxygen saturation, heart rate and 2-lead ECG throughout the CMR scan. Systemic blood pressure was periodically checked at every minute during adenosine stress and 15 min after the end of the infusion. The monitor tracing during the CMR scan does not allow for identification of 1^st ^or Wenckebach 2^nd ^degree AV block and therefore only advanced degrees of AV block (Mobitz II 2^nd ^degree and 3^rd ^degree) were noted. Each subject was questioned during and immediately after termination of adenosine infusion, specifically for the occurrence of the following symptoms: shortness of breath or dyspnea, chest pain and other minor symptoms (flushing, nausea, headache). At least two clinicians trained in cardiopulmonary resuscitation and CMR evacuation procedures were present during the adenosine infusion.

### Adenosine infusion protocol

All patients initially had the standard adenosine (Adenoscan^®^, Sanofi-Synthelabo) infusion dose of 140 mcg/kg/min for at least 3 minutes through an antecubital vein using a syringe pump (Graseby^® ^3500). If after 3 minutes of continuous infusion at the standard rate, the haemodynamic response to adenosine was inadequate (heart rate increase < 10 beats per minute or systolic blood pressure decrease < 10 mmHg, and minimal or no reported side effects from the patient) then the infusion rate was increased to 170 mcg/kg/min for a further 2 minutes. The absence or minimal presence of classical adenosine side effects was not a criterion to increase the dose if an adequate haemodynamic response was evidenced. If still patients failed to show evidence of peripheral vasodilatation then the infusion was increased to a maximum of 210 mcg/kg/min for a further 2 minutes. The infusion was discontinued if patients developed persistent or symptomatic 3^rd ^degree AV block, severe hypotension (systolic blood pressure *<*90 mmHg) or bronchospasm. The attending physicians had aminophylline for adenosine receptor antagonism and nitroglycerine for persistent chest pain readily available, and a fully equipped resuscitation trolley with defibrillator was easily accessible.

### CMR protocol

All CMR examinations were performed with subjects in a supine position on a 1.5 MR Tesla (Siemens Avanto, Erlangen, Germany) with a 32-element phased-array coil. During the last minute of adenosine infusion a gadolinium-based contrast agent (Gadodiamide, Omniscan^®^, GE Healthcare or Gadoterate meglumine, Dotarem^®^, Guerbet S.A.) was administered intravenously at 0.075 mmol/kg body weight (injection rate 4 ml/s), followed by a 20 ml saline flush at the same rate. Perfusion imaging was performed every cardiac cycle during the first pass, using a T_1_-weighted fast (spoiled) gradient echo sequence (echo time 1.05 ms, repetition time 2 ms, saturation recovery time 100 ms, voxel size 2.3 × 2.8 × 10 mm; flip angle 12°). Three or four short-axis slices, positioned from the base to the apex of the left ventricle, were obtained. The same imaging sequence was repeated at least 10 minutes later without adenosine to obtain perfusion images at rest. For assessment of left ventricular function, steady-state free-precession cine images (TE/TR 1.1/2.6 ms, voxel size 2.0 × 2.0 × 7 mm, flip angle 55°) were acquired in three long-axis views, and a short-axis stack to obtain coverage of the entire left ventricle. Analysis of left ventricular function was performed with Argus Syngo MR software (version B15, Siemens Healthcare, Erlangen, Germany) using the short-axis SSFP images as previously described [7]. The following left ventricular parameters were thereby determined: end-diastolic volume, end-systolic volume, ejection fraction and myocardial mass.

### Coronary angiography

Based on clinical criteria, a proportion of patients underwent coronary angiography. The decision to proceed to coronary angiography on the basis of an abnormal perfusion CMR scan was left at the discretion of the referring consultant cardiologist. Significant CAD was defined visually as the presence of at least one stenosis of > 50% diameter in any of the main epicardial coronary arteries or their branches with a diameter of ≥ 2 mm.

### Statistical analysis

Continuous variables are expressed as mean ± standard deviation. Categorical variables are expressed as counts and percentages. All continuous variables were found to be normally distributed by the Kolmogorov-Smirnov test. Differences in continuous variables between the standard- and high-dose groups were assessed by independent student's t-test. One-way analysis of variance (ANOVA) followed by post-hoc Bonferroni analysis was used to compare the haemodynamic response to stress in the high-dose group at several time points (baseline, standard dose and maximum infusion rate). Comparisons of categorical variables between groups were performed by *χ*^2 ^test or Fisher's exact test as appropriate. We used binary logistic regression analysis to identify potential predictors of inadequate response to standard adenosine dose. Receiver operator characteristic analysis was used to identify cut-offs for continuous variables such as age and ejection fraction. Based on those cut-off values, ejection fraction and age were entered into the regression model as dichotomous variables. A probability value of p < 0.05 was considered significant and two-tailed p values were used for all statistics. Statistical analyses were performed using MedCalc for Windows, version 11.3 (MedCalc Software, Mariakerke, Belgium) and the SPSS Statistics software (version 17.0; SPSS Inc., Chicago, Ill., USA).

## Results

### Patient characteristics

**Figure 1:**
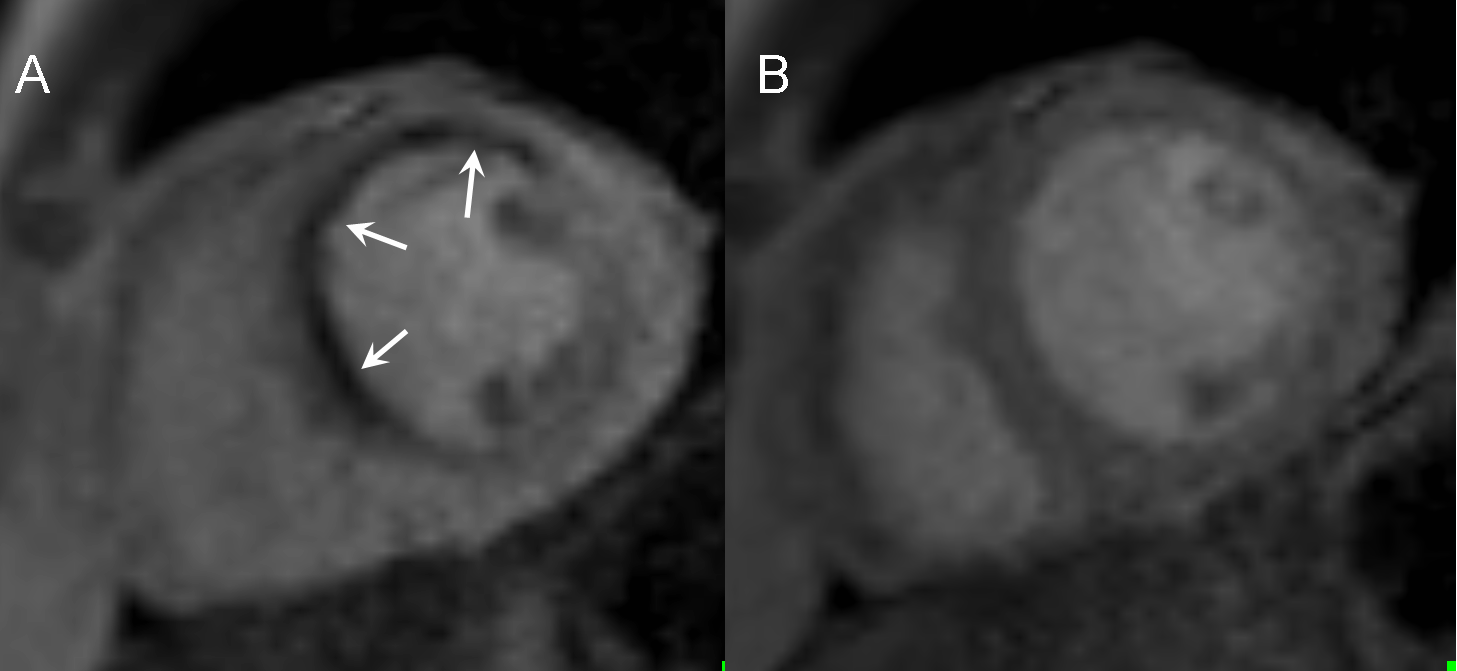
**A representative example from a CMR perfusion scan during high dose adenosine stress**. This is a 69-year-old diabetic patient with a significant stenosis of the left anterior descending coronary artery. Note the marked perfusion defect in the septum and anterior wall (arrows). The resting perfusion scan (panel B) is normal and shows homogeneous enhancement in all myocardial regions.

Data were collected from 98 consecutive subjects (mean age 58 ± 14 years, range 17-84 years) of whom 66 (67%) were men. Of the 98 subjects enrolled, 53 (54%) had a coronary angiogram. Of these 53 patients undergoing coronary angiography, 26 (49%) were found to have significant CAD: 20 single-vessel and 6 multi-vessel disease. The majority of our patients had undergone coronary angiography before the CMR scan. Specifically, 36 patients (68%) had a coronary angiogram before the CMR scan and 17 after the scan (32%). Of those 53 patients who underwent coronary angiography, 13 (25%) had normal perfusion on CMR, 32 (60%) had perfusion defects at stress and 8 (15%) had defects at stress corresponding to LGE without evidence of peri-infarct ischaemia. Table 1 shows the baseline characteristics of the subjects, and figure 1 shows a typical example from a first pass perfusion study.

**Table 1:** Characteristics of Study Population

	Standard dose group (n = 80)	High dose group (n = 18)	p-value
Age	56 ± 14	67 ± 10	0.001
Male	53 (66%)	13 (72%)	0.63
Height	172 ± 10	171 ± 11	0.89
Weight	84 ± 16	84 ± 19	0.98
BSA	1.99 ± 0.21	1.99 ± 0.28	0.95
Caffeine < 12 hours prior to scan	4 (5%)	2 (11%)	0.31
Hypertension	34 (43%)	7 (39%)	0.78
Diabetes	4 (5%)	4 (22%)	0.036
Hyperlipidaemia	35 (44%)	9 (50%)	0.63
Family history of CAD	12 (15%)	2 (11%)	1.00
Current Smoking	5 (6%)	0 (0%)	0.58
Coronary angiography	42 (53%)	11 (61%)	0.54
Single vessel CAD	14 (33%)	6 (38%)	0.49
Multi-vessel CAD	2 (5%)	4 (22%)	0.013
End-diastolic volume (ml)	157 ± 46	183 ± 61	0.10
End-systolic volume (ml)	59 ± 38	99 ± 62	0.018
Ejection fraction (%)	65 ± 13	52 ± 19	0.007
Mass index (gr/m^2^)	71 ± 21	72 ± 18	0.79
B-blockers	22 (28%)	6 (33%)	0.62
Ca-inhibitors	13 (16%)	4 (22%)	0.51
ACE-inhibitors	32 (40%)	9 (50%)	0.44
Statins	32 (40%)	9 (50%)	0.44
Aspirin	22 (28%)	8 (44%)	0.16

Values are mean ± standard deviation or number (percent).

ACE, angiotensin converting enzyme; BSA, body surface area; CAD, coronary artery disease

### Haemodynamic response to standard dose adenosine

Overall, 80 patients (82%) had an adequate response to the standard adenosine infusion rate of 140 mcg/kg/min with a significant increase in heart rate (p < 0.001) and rate pressure product (p < 0.001). There were minimal changes in systolic or diastolic blood pressure measurements. The mean duration of adenosine infusion was 246 ± 35 seconds (Table 2).

**Table 2:** Haemodynamic Parameters at Rest and During Adenosine Stress

Standard dose Adenosine (n = 80)				High dose Adenosine (n = 18)			
	**Rest**	**Peak**	**p-value**	**Rest**	**140 μg/kg/min**	**Peak**	**p-value**
HR(bpm)	70 ± 13	96 ± 16	< 0.001	66 ± 12	75 ± 11	81 ± 18*	0.011
SBP (mmHg)	137 ± 18	139 ± 19	0.21	139 ± 23	134 ± 24	132 ± 22	0.68
DBP (mmHg)	80 ± 12	81 ± 12	0.55	82 ± 12	79 ± 11	77 ± 14	0.57
RPP	9639 ± 2399	13304 ± 2781	< 0.001	8975 ± 1909	9945 ± 1694	10672 ± 2787	0.07

* p < 0.05 vs. rest

DBP, diastolic blood pressure; HR, heart rate; RPP, rate pressure product; SBP, systolic blood pressure

### Haemodynamic response to high dose adenosine

Eighteen patients (18%) failed to show signs of adequate haemodynamic response during standard-dose adenosine infusion. Following an increase in the dose of adenosine (up to 170 mcg/kg/min in 6 patients; and up to 210 mcg/kg/min in 12 patients) 16 out of these 18 patients showed an improved haemodynamic response with greater rise in heart rate (p = 0.011) and a mild drop in systolic blood pressure (p = 0.57). There was a trend towards a significant increase to rate pressure product at maximum adenosine rate compared to baseline (p = 0.07). The mean duration of adenosine infusion was significantly longer for the high-dose group (387 ± 46 seconds; p < 0.001) compared to the standard dose group (246 ± 35 seconds). Table 2 shows the haemodynamic effects of adenosine in both groups. Of the 80 patients who received standard dose adenosine 50 patients (63%) had abnormal perfusion on CMR (defined as any defect at stress irrespective of the LGE findings). Similarly, of the 18 patients who received high dose adenosine 10 (56%) patients had abnormal perfusion on CMR (p = 0.6).

### Adverse events

There were no deaths, myocardial infarctions, or episodes of bronchospasm or pulmonary edema in our cohort as a result of the adenosine stress. Transient advanced AV block (Mobitz II 2^nd ^degree and 3^rd ^degree) occurred in 3 subjects (8%), of whom 2 had the high-dose adenosine infusion and 1 the standard dose (p = 0.07). There were no sustained episodes of advanced AV block. More patients complained of chest pain/tightness in the high-dose group (p = 0.009), but these were short-lived, and sublingual nitrate administration was not required in any patients. There were no significant differences in the incidence of other common adenosine adverse effects such as shortness of breath, headache or flushing between the two groups. All these symptoms resolved shortly after termination of adenosine infusion and no aminophylline reversal was required. The adverse effects of adenosine in our cohort are summarised in Table 3.

**Table 3:** Adverse Effects of Adenosine

	Standard dose Adenosine (n = 80)	High dose Adenosine (n = 18)	p-value
Chest pain	23 (29%)	11 (61%)	0.009

Shortness of breath	19 (24%)	6 (33%)	0.40

Flushing, headache or dizziness	29 (37%)	9 (50%)	0.28

Mobitz II 2^nd ^or 3^rd ^degree atrioventricular block	1 (1%)	2 (11%)	0.09

Angina requesting sublingual nitrates	0 (0%)	0 (0%)	-

Scan abandoned during adenosine on subject's request	0 (0%)	0 (0%)	-

### Predictors of non-response to standard adenosine dose

**Figure 2:**
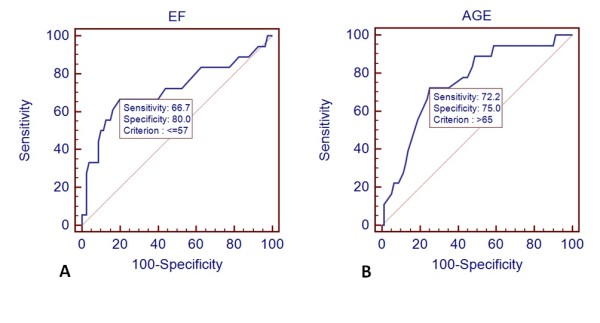
**Receiver-operator characteristic analyses to define cut-off values for age (panel A) and ejection fraction-EF (panel B) that are predictors of inadequate response to standard dose adenosine**.

Patients who showed an inadequate haemodynamic response to standard adenosine infusion rate were older and more often had a history of diabetes mellitus. Moreover, non-responders to standard adenosine dose had significantly lower ejection fractions compared to patients in the standard infusion group (Table 1). There were no differences between the two groups with reference to previous caffeine intake (p = 0.31). Receiver operator characteristic analysis showed that an age cut-off ≥ 65 years had 72% sensitivity and 75% specificity (area under the curve 0.753 ± 0.06, p = 0.0001) to predict inadequate response to adenosine at the standard infusion dose of 140 μg/kg/min (Figure 2). Similarly, an ejection fraction cut-off of < 57% had 67% sensitivity and 80% specificity (area under the curve 0.717 ± 0.08, p = 0.0083) to identify non-responders (Figure 2). Stepwise binary logistic regression analysis showed that age ≥ 65 years (OR 6.4-95% CI 1.9-22.0-p = 0.0032) and EF< 57% (OR 7.6-95% CI 2.2-25.4-p = 0.0011) were the two independent predictors of inadequate haemodynamic response to standard adenosine dose.

## Discussion

The present study is the first to demonstrate the feasibility and safety of high-dose adenosine in patients with known or suspected CAD undergoing stress perfusion CMR. Our findings suggest that a substantial number of patients (18% in our cohort) do not show a sufficient haemodynamic response to adenosine stress. Independent predictors of an inadequate response to the standard adenosine dose of 140 mcg/kg/min were: age above 65 years and ejection fraction less than 57%. The attenuated response to adenosine can be surmounted by increasing adenosine dose up to 210 mcg/kg/min without significant adverse events. Our study has important clinical implications because a reduced peripheral haemodynamic response to standard dose intravenous administration of adenosine may reflect similarly inadequate effects on coronary vasodilatation, thereby affecting the diagnostic reliability of CMR perfusion imaging.

Adenosine is a powerful vasoactive substance. Activation of cardiac A_2A _and A_2B _adenosine receptors dilates the coronary and peripheral arterial beds (with the exception of renal glomerular afferent arteriole), increases myocardial blood flow, and causes sympatho-excitation [4]. These effects cause mild decrease in blood pressure and baroreceptor-mediated reflex tachycardia [8,9]. The majority of patients reach near maximal coronary vasodilatation and show signs of sufficient peripheral vasodilatory response after 2-3 minutes of adenosine infusion at the standard dose of 140 mcg/kg/min. However, as previous studies have demonstrated, some patients show reduced vasodilator response to the standard dose [4-6].

In our study 18% of patients showed inadequate peripheral haemodynamic response to the standard infusion dose. Similarly, Wilson et al. measured coronary blood flow invasively using Doppler and found that 16% of their subjects who received adenosine at 140 mcg/kg/min did not achieve maximal coronary hyperaemia as compared to that produced with papaverine [4]. The mechanism of this phenomenon is not well understood, but it is interesting that similar differences in adenosine responsiveness have also been reported in dogs [10]. Impairment of the baroreceptor reflex, resulting in decreased vascular reactivity in response to vasodilator stimuli is a possible cause of reduced heart rate response. As previously shown, diabetic or elderly patients have attenuated reflex tachycardia in response to vasodilator-induced decreased blood pressure, possibly attributable to autonomic dysfunction [5,11]. Indeed, our study patients who showed reduced haemodynamic response were significantly older and were more likely to have history of diabetes mellitus than those with normal response to standard adenosine. Moreover, a left ventricular ejection fraction of less than 57% was also associated with inadequate peripheral haemodynamic response. This may be an effect of the already increased sympathetic nervous activity known to be present in heart failure. It is well known that patients with heart failure have high resting peripheral vasomotor tone which makes them less responsive to the vasodilator properties of adenosine [12,13]. There is also an association between high catecholamine levels and endogenous adenosine formation which acts as a negative feedback system in patients with heart failure [13]. Lastly, besides physiological considerations, genetic factors are likely to play an important role in determining response to adenosine. Potency of adenosine is strongly influenced by the number of adenosine receptors expressed in myocardial cells, the predominant type of receptor expressed, as well as the type of response that is measured from adenosine stimulation [14-17].

Our data suggest that there is a clear dose-response relation and when patients fail to show adequate vasomotor responsiveness, a further increase in adenosine dose can induce signs of improved peripheral hemodynamic effect. This dose-dependent vasodilator effect is also supported by previous studies using intravenous or intracoronary doses of adenosine [18-21]. Reyes and colleagues have used a high-dose adenosine protocol (210 mcg/kg/min) to overcome caffeine antagonism in patients undergoing myocardial perfusion scintigraphy [22]. In total, 18 patients received the high dose after 200 mg of caffeine intake. The increased adenosine dose was well tolerated and was effective to surmount the inhibitory effect of caffeine. Our study is the first to use high dose adenosine in patients undergoing perfusion CMR imaging who fail to show an adequate cardiovascular response, irrespective of caffeine use. Although our patient cohort is small, all 18 patients who received high dose adenosine infusion tolerated it well without significant adverse clinical events. We did not measure caffeine levels in our patient population and, therefore, recent caffeine intake, despite the clear instructions to avoid it for 24 hours, cannot be excluded. However, as previously shown, even patients who consume coffee 1-2 hours before the scan and reach peak levels of caffeine during the infusion of adenosine may demonstrate similar hemodynamic response to stress with patients who had no caffeine [22-24]. Therefore, we believe that the insufficient response to adenosine stress which 18 of our patients showed is unlikely to be due to previous caffeine intake.

Previous studies looking into the relation between the peripheral hemodynamic actions of adenosine and its coronary vasodilator effect have shown that despite a lack of peripheral response to adenosine, coronary vasodilatation remains adequate for the purpose of myocardial perfusion imaging [25]. This observation would suggest that a further increase to adenosine dose in order to induce better systemic and coronary vasodilatation to non-responders to standard dose is not needed. However, there are important differences between our study and the aforementioned study by Mishra and colleagues. First of all, we prospectively defined 'non-responders' to standard adenosine dose (HR rise < 10 bpm, SBPdrop < 10 mmHg) whereas Mishra and colleagues retrospectively looked into the lower tertile which had an average HR change of 13 bpm. Notwithstanding this, the patients in the lower tertile had lower hyperemic myocardial blood flow and higher coronary resistance compared to the patients in the intermediate (average HR increase 25 bpm) and upper tertiles (average HR change 37 bpm) [25]. This observation supports our hypothesis that inadequate peripheral hemodynamic response to adenosine might indicate reduced coronary vasodilatation and slightly increased coronary resistance. Lastly, Mishra and colleagues did not test the peripheral and coronary haemodynamic effects of a high dose infusion protocol.

This study did not assess any potential advantage of high-dose adenosine perfusion CMR on the diagnostic performance of this technique in patients with reduced haemodynamic response to standard adenosine dose. To perform such a study the same cohort of patients with blunted vasodilatory response would need to undergo stress perfusion CMR twice using the standard- and the high-dose adenosine infusion with not only visual but also quantitative measurements of myocardial blood flow. Nevertheless, our aim was to show feasibility and initial safety data of the high-dose adenosine infusion protocol and not to assess the potential added clinical value of such an approach. Stress echocardiography using a high dose adenosine protocol has been shown to have incremental diagnostic value mainly by increasing sensitivity in patients with single-vessel disease [21]. It is conceivable that this may also be true for stress perfusion CMR, but larger scale studies are needed to confirm our initial observations on safety of the proposed high-dose protocol and assess its diagnostic performance in everyday practice.

## Conclusions

About one fifth of patients undergoing stress perfusion CMR show inadequate haemodynamic response to adenosine infusion at the standard dose of 140 mcg/kg/min, for which age > 65 years and ejection fraction < 57% were independent predictors. A high-dose protocol (up to 210 mcg/kg/min) improves the peripheral haemodynamic response to adenosine, suggesting better coronary vasodilatation. Our initial experience with a high dose adenosine infusion suggests it is well tolerated and safe, although further confirmation is needed in larger scale studies which will test also the clinical utility of this protocol.

## Competing interests

All authors declare that they have no conflicts of interest related to the contents of this manuscript.

## Authors' contributions

TDK: conceived the study, performed and analysed CMR scans, drafted the manuscript, did statistical analysis. NN: performed and analysed CMR scans, helped with drafting the manuscript, data collection and analysis. JMF: performed CMR scans. CH: performed and analysed CMR scans, critically revised the manuscript. SGM: critically revised the manuscript. SN: critically revised the manuscript, guarantor of this work. All authors read and approved the final manuscript.

## References

[B1] Gerber BL Raman SV Nayak K Epstein FH Ferreira P Axel L Kraitchman DL Myocardial first-pass perfusion cardiovascular magnetic resonance: history, theory, and current state of the art J Cardiovasc Magn Reson 2008 10 18 10.1186/1532-429X-10-18 18442372 PMC2387155

[B2] Christiansen JP Karamitsos TD Myerson SG Francis JM Neubauer S Stress Perfusion Imaging Using Cardiovascular Magnetic Resonance: A Review Heart Lung Circ 2010 10.1016/j.hlc.2010.08.008 20869310

[B3] Karamitsos TD Arnold JR Pegg TJ Cheng AS van Gaal WJ Francis JM Banning AP Neubauer S Selvanayagam JB Tolerance and safety of adenosine stress perfusion cardiovascular magnetic resonance imaging in patients with severe coronary artery disease Int J Cardiovasc Imaging 2009 25 277 283 10.1007/s10554-008-9392-3 19037746

[B4] Wilson RF Wyche K Christensen BV Zimmer S Laxson DD Effects of adenosine on human coronary arterial circulation Circulation 1990 82 1595 1606 2225364 10.1161/01.cir.82.5.1595

[B5] Johnston DL Hodge DO Hopfenspirger MR Gibbons RJ Clinical determinants of hemodynamic and symptomatic responses in 2,000 patients during adenosine scintigraphy Mayo Clin Proc 1998 73 314 320 10.4065/73.4.314 9559034

[B6] Abidov A Hachamovitch R Hayes SW Ng CK Cohen I Friedman JD Germano G Berman DS Prognostic impact of hemodynamic response to adenosine in patients older than age 55 years undergoing vasodilator stress myocardial perfusion study Circulation 2003 107 2894 2899 10.1161/01.CIR.0000072770.27332.75 12796141

[B7] Karamitsos TD Hudsmith LE Selvanayagam JB Neubauer S Francis JM Operator induced variability in left ventricular measurements with cardiovascular magnetic resonance is improved after training J Cardiovasc Magn Reson 2007 9 777 783 10.1080/10976640701545073 17891615

[B8] Cerqueira MD Verani MS Schwaiger M Heo J Iskandrian AS Safety profile of adenosine stress perfusion imaging: results from the Adenoscan Multicenter Trial Registry J Am Coll Cardiol 1994 23 384 389 10.1016/0735-1097(94)90424-3 8294691

[B9] Biaggioni I Olafsson B Robertson RM Hollister AS Robertson D Cardiovascular and respiratory effects of adenosine in conscious man. Evidence for chemoreceptor activation Circ Res 1987 61 779 786 3677336 10.1161/01.res.61.6.779

[B10] Olsson RA Khouri EM Bedynek JL Jr McLean J Coronary vasoactivity of adenosine in the conscious dog Circ Res 1979 45 468 478 476869 10.1161/01.res.45.4.468

[B11] Czernin J Muller P Chan S Brunken RC Porenta G Krivokapich J Chen K Chan A Phelps ME Schelbert HR Influence of age and hemodynamics on myocardial blood flow and flow reserve Circulation 1993 88 62 69 8319357 10.1161/01.cir.88.1.62

[B12] Hasking GJ Esler MD Jennings GL Burton D Johns JA Korner PI Norepinephrine spillover to plasma in patients with congestive heart failure: evidence of increased overall and cardiorenal sympathetic nervous activity Circulation 1986 73 615 621 3948363 10.1161/01.cir.73.4.615

[B13] Triposkiadis F Karayannis G Giamouzis G Skoularigis J Louridas G Butler J The sympathetic nervous system in heart failure physiology, pathophysiology, and clinical implications J Am Coll Cardiol 2009 54 1747 1762 10.1016/j.jacc.2009.05.015 19874988

[B14] Belardinelli L Shryock JC Snowdy S Zhang Y Monopoli A Lozza G Ongini E Olsson RA Dennis DM The A2A adenosine receptor mediates coronary vasodilation J Pharmacol Exp Ther 1998 284 1066 1073 9495868

[B15] Mizuno M Kimura Y Tokizawa K Ishii K Oda K Sasaki T Nakamura Y Muraoka I Ishiwata K Greater adenosine A(2A) receptor densities in cardiac and skeletal muscle in endurance-trained men: a [11C]TMSX PET study Nucl Med Biol 2005 32 831 836 10.1016/j.nucmedbio.2005.07.003 16253807

[B16] Mustafa SJ Morrison RR Teng B Pelleg A Adenosine receptors and the heart: role in regulation of coronary blood flow and cardiac electrophysiology Handb Exp Pharmacol 2009 161 188 full_text 19639282 10.1007/978-3-540-89615-9_6 PMC2913612

[B17] Talukder MA Morrison RR Jacobson MA Jacobson KA Ledent C Mustafa SJ Targeted deletion of adenosine A(3) receptors augments adenosine-induced coronary flow in isolated mouse heart Am J Physiol Heart Circ Physiol 2002 282 H2183 2189 12003827 10.1152/ajpheart.00964.2001 PMC10775950

[B18] Lopez-Palop R Saura D Pinar E Lozano I Perez-Lorente F Pico F Valdez M Adequate intracoronary adenosine doses to achieve maximum hyperaemia in coronary functional studies by pressure derived fractional flow reserve: a dose response study Heart 2004 90 95 96 10.1136/heart.90.1.95 14676256 PMC1768040

[B19] Lindstaedt M Bojara W Holland-Letz T Yazar A Fadgyas T Muller L Mugge A Germing A Adenosine-induced maximal coronary hyperemia for myocardial fractional flow reserve measurements: comparison of administration by femoral venous versus antecubital venous access Clin Res Cardiol 2009 98 717 723 10.1007/s00392-009-0056-7 19685258

[B20] Kern MJ Deligonul U Tatineni S Serota H Aguirre F Hilton TC Intravenous adenosine: continuous infusion and low dose bolus administration for determination of coronary vasodilator reserve in patients with and without coronary artery disease J Am Coll Cardiol 1991 18 718 729 10.1016/0735-1097(91)90795-B 1869735

[B21] Djordjevic-Dikic AD Ostojic MC Beleslin BD Stepanovic J Petrasinovic Z Babic R Stojkovic SM Stankovic G Nedeljkovic M Nedeljkovic I Kanjuh V High dose adenosine stress echocardiography for noninvasive detection of coronary artery disease J Am Coll Cardiol 1996 28 1689 1695 10.1016/S0735-1097(96)00374-9 8962553

[B22] Reyes E Loong CY Harbinson M Donovan J Anagnostopoulos C Underwood SR High-dose adenosine overcomes the attenuation of myocardial perfusion reserve caused by caffeine J Am Coll Cardiol 2008 52 2008 2016 10.1016/j.jacc.2008.08.052 19055993

[B23] Zoghbi GJ Htay T Aqel R Blackmon L Heo J Iskandrian AE Effect of caffeine on ischemia detection by adenosine single-photon emission computed tomography perfusion imaging J Am Coll Cardiol 2006 47 2296 2302 10.1016/j.jacc.2005.11.088 16750699

[B24] Aqel RA Zoghbi GJ Trimm JR Baldwin SA Iskandrian AE Effect of caffeine administered intravenously on intracoronary-administered adenosine-induced coronary hemodynamics in patients with coronary artery disease Am J Cardiol 2004 93 343 346 10.1016/j.amjcard.2003.10.017 14759387

[B25] Mishra RK Dorbala S Logsetty G Hassan A Heinonen T Schelbert HR Di Carli MF Quantitative relation between hemodynamic changes during intravenous adenosine infusion and the magnitude of coronary hyperemia: implications for myocardial perfusion imaging J Am Coll Cardiol 2005 45 553 558 10.1016/j.jacc.2004.10.064 15708703

